# Supplementary material for: Identification of photoperiod-induced specific miRNAs in the adrenal glands of Sunite sheep (Ovis aries)
Source: Front Vet Sci. 2022 Jul 22;9:888207. doi: 10.3389/fvets.2022.888207 (PMC9354845; doi:10.3389/fvets.2022.888207)
Supplement: Supplementary file 1 [file Data_Sheet_1.ZIP › Supplementary file/miRDeep2 file.zip!/miRDeep2 file/Know_Structure/SPLP42A3/oar-mir-544.pdf]

miRBase precursor : oar-mir-544
Total read count : 301
oar-miR-544-5p read count : 278
oar-miR-544-3p read count : 12
remaining reads : 11

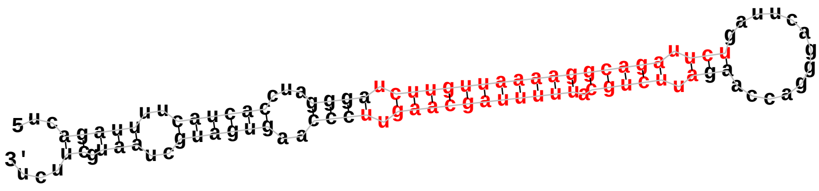

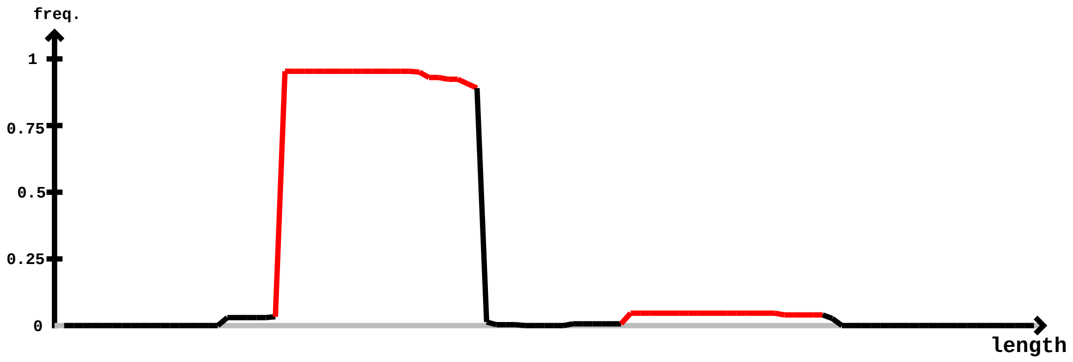

oar-miR-544-5p

oar-miR-544-3p

```
5'- ucagauuuucaucaccuagggaucuuguuaaaaggcagauucugauucagggaccaagauucugcauuuuuagcaaguucccaagugaugcuaaugcuucu   -3'    exp
    ..(((((..((((((...((((.((((((((((((((((.(((.............))).))))).)))))))))))).))))..))))))..))).))...    reads     mm     sample
    ................uagggaucuuguuaaaaggc.................................................................    1         0      seq
    ................uagggaucuuguuaaaaggca................................................................    6         0      seq
    ................uagggaucuuguuaaaaggcaga..............................................................    1         0      seq
    ................uagggauUuuguuaaaaggcaga..............................................................    1         1      seq
    .....................aucuuguuaaaaggcagauucu..........................................................    1         0      seq
    ......................ucuuguuaaaaggcagauu............................................................    4         0      seq
    ......................ucuuguuaaaaggcagauA............................................................    1         1      seq
    ......................ucuuguuaaaaggAagauuc...........................................................    1         1      seq
    ......................ucuuguuaaaaggcagauuc...........................................................    4         0      seq
    ......................ucuuguuaaaaggcagauAcu..........................................................    1         1      seq
    ......................ucuuguuaaaaggcagauucA..........................................................    15        1      seq
    ......................ucuuCuuaaaaggcagauucu..........................................................    1         1      seq
    ......................ucuuguuaaaaggcagauucu..........................................................    238       0      seq
    ......................ucuuAuuaaaaggcagauucu..........................................................    1         1      seq
    ......................ucuuguuCaaaggcagauucu..........................................................    1         1      seq
    ......................Ccuuguuaaaaggcagauucu..........................................................    5         1      seq
    ......................ucuuguuaGaaggcagauucu..........................................................    1         1      seq
    ......................ucuuguuaaaaggcagauucuA.........................................................    3         1      seq
    ......................ucuuguuaaaaggcagauucugauu......................................................    1         0      seq
    ....................................................accaagauucugcauuuuuagc...........................    2         0      seq
    ..........................................................auucugcauuuuuagcaaguu......................    4         0      seq
    ..........................................................auucugcauuuuuagcaaguuU.....................    3         1      seq
    ..........................................................auucugcauuuuuagcaaguuc.....................    5         0      seq
```
